# The QT Interval Dynamic in a Human Experimental Model of Controlled Heart Rate and QRS Widening

**DOI:** 10.3390/jcm8091417

**Published:** 2019-09-09

**Authors:** Santiago Colunga, Remigio Padrón, Daniel García-Iglesias, José Manuel Rubín, Diego Pérez, Raquel del Valle, Pablo Avanzas, César Morís, David Calvo

**Affiliations:** 1 Cardiology Department, Hospital Universitario Central de Asturias, 33011 Oviedo, Spain (S.C.) (R.P.) (D.G.-I.) (J.M.R.) (D.P.) (R.d.V.) (P.A.) (C.M.); 2 Instituto de Investigación Sanitaria del Principado de Asturias, 33011 Oviedo, Spain; 3 Department of Medicine, Universidad Católica de Murcia, 30107 Guadalupe, Murcia, Spain

**Keywords:** QT interval, QRS width, correction methods

## Abstract

**Background:** there is increasing interest for computing corrected QT intervals in patients with prolonged depolarization. We aimed to analyze the effect of prolonged QRS in the QT and in the diagnostic accuracy of frequency-correction. **Methods and Results:** in 28 patients admitted for self-expanding aortic valve implantation, sequential pacing was performed in the AAI mode in two different phases: before and immediately after the release of the prosthesis. We evaluated the accuracy of the Bazett, Fridericia, Framingham and Hodges formulas with the reference of the QT at 60 bpm (QTc/deviation). The widening of the QRS was the main contributor to the QT prolongation (Pearson 0.79; CI95%: 0.75–0.84). Prolongation in other intervals (ST segment and T-wave) significantly contribute in the higher frequency range (*p* < 0.05). The Bazett’s formula displayed the highest QTc/deviation, while Framingham and Hodges retrieved the lowest QTc/deviation and the best fit (*p* < 0.001). In addition, the Bazett’s formula displayed the highest correlation between variations in the QTc/deviation and the widening of the QRS (Pearson coefficient −0.54; *p* < 0.001) in comparison with the Fridericia, Framingham and Hodges formulas (−0.51, −0.37 and −0.38 respectively; *p* < 0.001). There was also a linear effect of the heart rate in the QTc/deviation obtained with the Bazett’s formula (*p* = 0.015), not observed for other formulas. **Conclusions:** The prolonged depolarization of the ventricles introduces direct and linear prolongation in the QT interval, but also a non-linear distortion in cardiac repolarization that contributes for QT prolongation at the higher frequency range. The Bazett’s formula displays significantly higher sensitivity to prolongation of ECG intervals.

## 1. Introduction

The evaluation of the QT interval is critical as it can be altered by multiple factors that increase the risk of malignant arrhythmias [1]. The QT interval is also highly dynamic, displaying an adaptive behavior that is characteristically linked to the frequency of the heartbeat. Clinicians are well aware that as the heart rate increases, the QT interval is proportionally shortened and vice versa [1]. That is the reason why a variety of different formula have attempted frequency correction of the QT interval with different success in the clinic [2]. But less attention has been paid to the time for cardiac depolarization as a matter affecting variability of the QT intervals and accuracy of frequency-correction methods.

Today, there is increasing interest in developing appropriate methods for computing QT intervals in the patient with prolonged depolarization of the ventricles (increased QRS duration). Overall, a variety of seminal works provide with different methods that allow for correction of QT intervals with regard to the QRS width [3,4,5]. However, little is known about the mechanisms by which prolonged depolarization (i.e., bundle branch block) artifacts the QT interval duration and the linearity of the responses, which in turn introduce some uncertainty. In addition, frequency correction is still required. There is no evidence on the effect of QRS duration in the predictive capabilities of available formulas. All together, these make it important to address a comprehensive approach to dual correction (frequency plus QRS duration), by analyzing the relationship of the QRS width with: (i) the duration of the repolarization itself; and (ii) the diagnostic accuracy of methods for frequency correction. In the present work we demonstrate that there is a proportional prolongation of the QT interval with increased QRS duration, with other ECG intervals being significantly affected at the higher frequency range. The latter affects accuracy of different frequency correction methods, making some formulas more sensitive than others to the distortion introduced by prolonged depolarization.

## 2. Experimental Section

### 2.1. Patients and Experimental Protocol

An experimental protocol of controlled heart rate was conducted in 28 patients admitted to our hospital for implantation of a percutaneous self-expanding aortic valve (Medtronic Corevalve Evolut^®^) as previously reported [6]. Under conscious sedation, a dual-chamber transient pace-maker was inserted through the left femoral and right jugular veins to locate the atrial electrode at the right atrial appendage and the ventricular electrode at the right ventricular apex. Pacing was sequentially performed in the AAI mode within a range of 50 to 120 beats per minute (bpm). Each pacing burst consisted in continuous stimulation at a fixed frequency for at least 30 s to allow stabilization of ECG intervals. After each burst of pacing, the stimulation was stopped for 15 s and started at a new frequency (10 bpm up; Limited by Wenckeback point). The stimulation protocol was performed under continuous digital recording of the standard 12 lead ECG and stored for off-line analysis (1.2 KHz sample-rate; band-pass filtered 0.05–150 Hz; ArtisSensis©, Siemmens^®^). Patients gave informed consent and the ethic committee of our institution approved the research.

**Figure 1:**
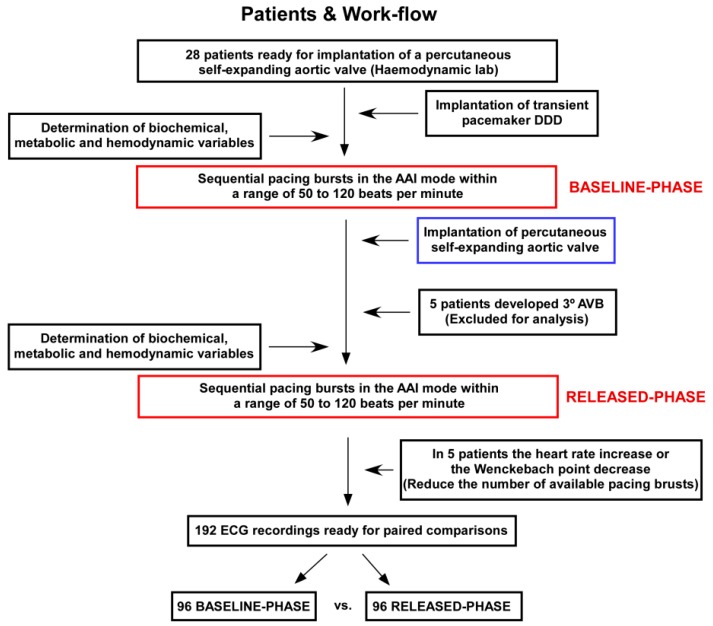
Flow-chart of patients included for analysis and experimental protocol.

The pacing protocol was conducted in two different phases (Figure 1; Flowchart): (i) before any intervention on the native aortic valve (baseline-phase); and (ii) immediately after the release of the percutaneous prosthesis (released-phase). The patient had to be in sinus rhythm throughout the pacing protocol. Investigators paid attention to widening of the QRS interval after the valve release, which is a common feature of the percutaneous self-expanding aortic valve implantation procedure [6,7]. Thus, our protocol provided the ability to explore the QT behavior under controlled frequency ranges and within variable times for depolarization of the ventricles. Other possible confounding factors affecting the QT duration were analyzed by serial determinations of biochemical, metabolic and hemodynamic conditions. For that purpose, we performed repeated analytical determinations at the baseline-phase and the released-phase including serial determinations of ion K, ion Ca, ion Na, ion Cl, pCO_2_, pH, HCO3-, hemoglobin, lactate and glucose. In addition, body temperature and changes in the pressure recorded from the left ventricle cavity was continuously monitored and analyzed.

### 2.2. ECG Measurements, Frequency Correction and Definitions

**Figure 2:**
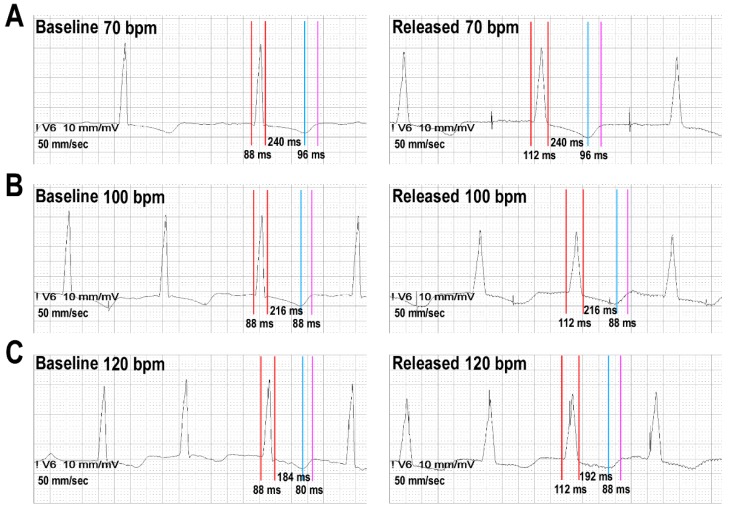
Example of measurements and behavior of ECG intervals.

Average QRS width and QT intervals were measured for each stimulation burst (using integrated calipers over the digital records; speed 100 mm per sec). Average was defined according to measurements in three consecutive cycles, after excluding the first 10 beats of each pacing burst to allow for stabilization of ECG intervals. The QT interval with better definition in the precordial leads (measurement of biphasic, flat T waves or prominent QT-U complexes was avoided whenever possible) was manually measured by the tangent method [8]. The QRS was measured from its begging to the J-point. For analytical purposes we divided the QRS-T complexes in: (i) the QRS interval, as a marker of cardiac depolarization; (ii) the interval from the ending of the QRS to the peak of the T wave (QRSend-Tpeak), as a marker of the plateau phase of cardiac myocytes and the beginning of repolarization; and (iii) the interval from the peak of the T wave to the end of the T wave (Tpeak-Tend), as marking the end of repolarization and a measure of the transmural dispersion of the repolarization. Examples of measurements are displayed in Figure 2. The changes in the QT interval between different phases of the pacing protocol were computed as δ-QT = (QT at released-phase) − (QT at baseline-phase). The same methodology was followed to compute the δ-QRS, the δ-QRSend-Tpeak and the δ-Tpeak-Tend.

Four formulas for frequency correction of the QT interval (QTc) were analyzed. Two non-linear formulas in which the heart rate (RR interval) is expressed in seconds: Bazett (QTcBZT) and Fridericia (QTcFRD); and two linear formulas in which the RR interval is expressed in milliseconds: Hodges (QTcHDG) and Framingham (QTcFRM). All these formulas are functions of two variables; the value of the QT interval and the RR interval summarized as follows:

- Bazett: [9] QTcBZT = QTc = QT/RR^1/2^
- Fridericia: [10] QTcFRD = QT/RR^1/3^
- Framingham: [11] QTcFRM = QTc = QT + 0.154 (1 − RR)
- Hodges: [12] QTcHDG = QT + 0.00175 ((60/RR) − 60)

Subsequently, we computed the deviation of the QTc (QTc_/deviation_) retrieved by each correction formula (BZT-QTc_/deviation_, FRD-QTc_/deviation_, FRM-QTc_/deviation_ and HDG-QTc_/deviation_ respectively) with regard to the QT measured at the frequency of 60 bxm (QT60). The QTc_/deviation_ was calculated for each phase of the pacing protocol and differences between them were computed as the δ-QTc_/deviation_ = QTc_/deviation_ during released-phase − QTc_/deviation_ during baseline-phase.

### 2.3. Statistics and Data Analysis

If normally distributed, continuous variables are reported as mean ± standard deviation (±SD) or followed by Confidence Interval 95% (CI95%). Categorical variables are reported as number and percentage. The Kolmogorov test was used to check normal distribution of variables. Due to their symmetry, equality in the variables behavior was checked by using unpaired and paired Student-Welch tests for the continuous variables. Pearson’s coefficient was used for measuring correlation between continuous variables. A paired ANOVA was used to test differences in multiple paired comparisons. We used a generalized linear model to test changes in the ECG intervals across sequential stages from 50 to 120 bpm during the pacing protocol. The Bonferroni’s correction was used for multiple comparisons.

Due to the data were collected in clusters (patients) and in order to handling the existing correlation in the ECG data within the same clusters, the general bootstrap algorithm was used for contrasting the equality between groups. Specifically, the naive bootstrap [13] and the general bootstrap algorithms [14] were used for confidence intervals and testing, respectively. In both cases, the resampling was made on the subjects. CI95% was also computed by resampling methods. Analyses were performed with R version 3.1 (www.r-project.org) and SPSS version 23 (SPSS Inc., Chicago, IL, USA). Statistical significance was established at *p* < 0.05.

## 3. Results

### 3.1. Patients and the Dynamic of ECG Intervals

We analyzed 28 consecutive patients, from whom a total of 282 pacing burst were performed (Figure 1). At the released-phase, five patients were excluded for analysis because they developed third degree atrio-ventricular block. In another five patients, the heart rate increased from the baseline-phase or the atrio-ventricular Wenckebach point decrease, thus decreasing the number of pacing bursts available for comparative analysis between phases. Overall, we recorded 192 ECG tracings from 23 patients, 96 ECG tracings of each phase (baseline-phase vs. released-phase) available for paired comparisons. The clinical characteristics of patients included for analysis are displayed in Table 1.

Comparative analysis of ECG measurements between phases are displayed in Table 2. In summary, the released-phase was followed by significant QRS widening (32.5 ms; CI95%: 31.5–33.6) and QT prolongation (35.4 ms; CI95%: 33.4–37.4). As expected, the magnitude of the QT interval linearly decreases from the lower to the highest frequency range (*p* < 0.001 both baseline-phase and released-phase). Table 3 shows the analysis of possible confounding factors affecting ECG intervals between phases. No clinically significant differences were observed in terms of biochemical or metabolic parameters. As expected, the telesystolic and telediastolic pressures recordings from the left ventricle significantly changed after the release of the prosthesis. However, the amount of change was not associated with the δ-QRS (Pearson coefficient −0.24; CI95%: −0.62–0.36, and 0.13; CI95%: −0.35–0.60 respectively with telesystolic and telediastolic pressure) nor with the δ-QT (Pearson coefficient −0.061; CI95%: −0.57–0.43 and 0.19; CI95%: −0.59–0.79 respectively).

**Figure 3:**
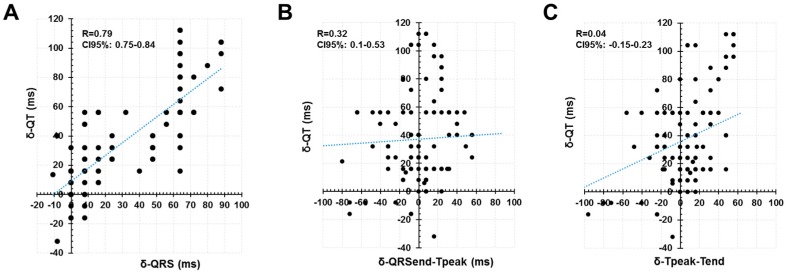
Correlation of δ-QT with regard to δ-QRS, δ- the interval from the ending of the QRS to the peak of the T wave (QRSend-Tpeak) and δ- the interval from the peak of the T wave to the end of the T wave (Tpeak-Tend). See text for discussion.

**Figure 4:**
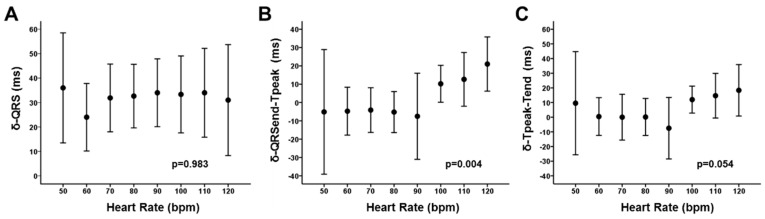
Heart rate dependence of δ-QRS, δ-QRSend-Tpeak and δ-Tpeak-Tend. See text for discussion.

In an attempt to analyze the role of different periods of cardiac depolarization and repolarization on the dynamic of the QT prolongation, we analyzed the behavior of the different segments within the QRS-T complexes that are considered to represent specific timings of the cardiac cycle. We observed a good fit between the δ-QT and the δ-QRS (Pearson coefficient 0.79; CI95%: 0.75–0.84). But the correlation was significantly lower with regard to the δ-QRSend-Tpeak (Pearson coefficient 0.32; CI95%: 0.1–0.53) and the δ-Tpeak-Tend (Pearson coefficient 0.04; CI95%: −0.15–0.23) (Figure 3). Thus, the widening of the QRS seemed to be the main contributor to the QT prolongation in our patients. However, we noticed significant effect of the heart rate as a modulator of the contribution of different segments to the final QT prolongation. Figure 4 summarizes the results and displays that the δ-QRS remain constant along the spectrum of the heart rate (up to 120 bpm; Panel A). Similarly, the behavior of the δ-QRSend-Tpeak (panel B) and the δ-Tpeak-Tend (panel C) displayed that for the frequencies below 100 bpm there is not significant prolongation between baseline-phase and released-phase. But they tended to increase for the higher frequency-range up to 120 bpm (*p* < 0.05 for both models). The latter is interpreted as the δ-QRSend-Tpeak and the δ-Tpeak-Tend having negligible (scattered) contribution to the δ-QT for the lower heart-rate, but seemed to be significant contributors at the higher heart-rate.

### 3.2. Performance of Frequency-Correction Formulas

**Figure 5:**
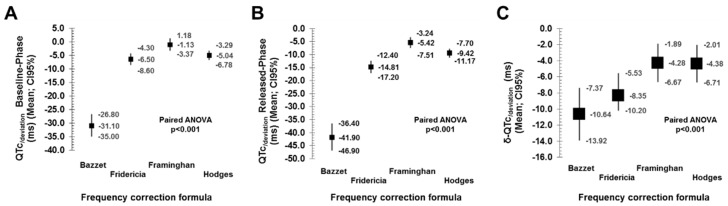
Comparative performance of the QTc_/deviation_. See text for discussion.

**Figure 6:**
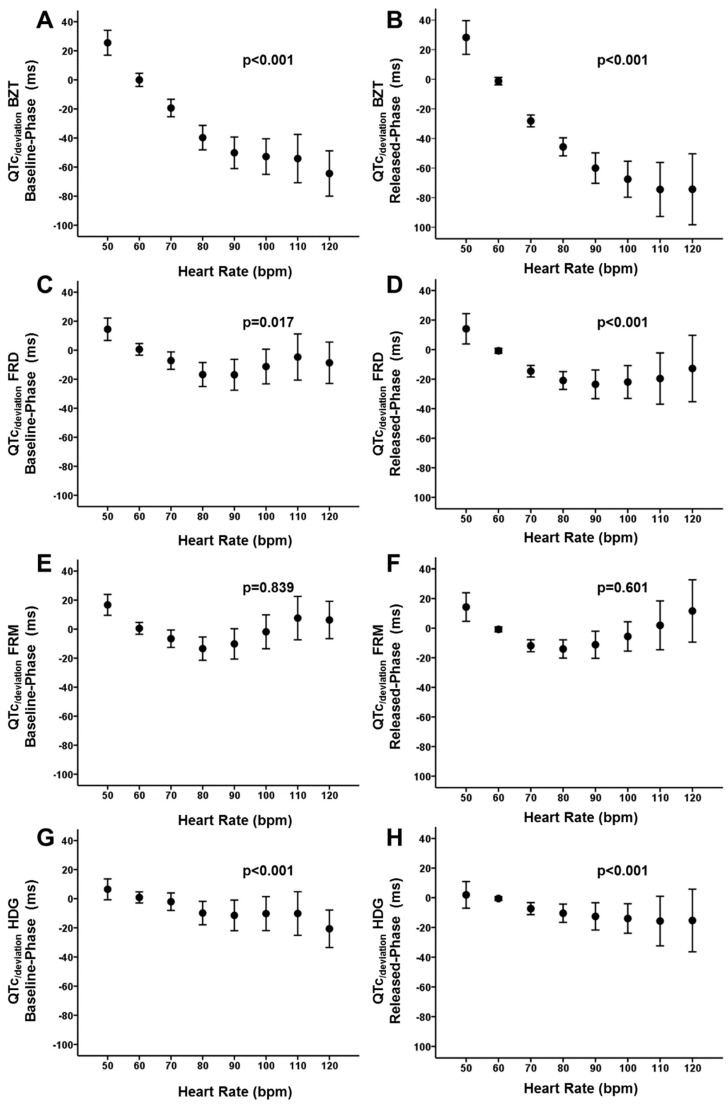
Heart rate dependence of the QTc_/deviation_. See text for discussion. BZT: Bazett formula. FRD: Fridericia formula. FRM: Framinghan. HDG: Hodges formula.

As previously described, we analyzed the accuracy of correction formulas to compute the QTc with the reference of the QT60 [9,10,11,12]. Table 4 and Figure 5 summarize the correlation coefficients between the QT60 and the corresponding QTc, and the QTc_/deviation_ obtained for each correction formula. It is observed that Bazett`s formula is the one that displayed the highest deviation and the lowest correlation. It was followed by the Fridericia, Framingham and Hodges formulas. The last two retrieved the lowest deviation and the best fit. Overall, those results are consistent during both phases of the protocol, either baseline-phase or released-phase (Figure 5, panels A and B). However, we noticed again a significant effect of the heart rate. Figure 6 displays quantitative analysis of the effect of heart rate on the QTc_/deviation_. Each panel from A to D represents cumulative data for Bazett, Fridericia, Framingham and Hodges formulas respectively. It is observed that for the Bazett’s formula the QTc_/deviation_ was highly dependent on the heart rate (*p* < 0.001, both baseline-phase and released-phase). As previously described [15], for the lowest frequency (50 bpm) the QTc tends to be overestimated while for the highest frequencies the QTc tends to be underestimated. The same pattern was observed for the Fridericia formula (*p* = 0.019 and *p* < 0.001, respectively for baseline-phase and released-phase). With the Hodges formula there was also a linear tendency displaying an overestimation at the highest frequencies, but not significant fitting errors seemed to occur at the lower frequency range (*p* < 0.001, both baseline-phase and released-phase). However, with the Framingham formula there was not any linear tendency observed (*p* = 0.829 and *p* = 0.601, respectively for baseline-phase and released-phase). The latter resembles a U-shape distribution of the QTc_/deviation_ when analyzed at the different heart rates, but most of the values remain close to 0 thus suggesting a more appropriate fitting and less heart rate dependence.

**Figure 7:**
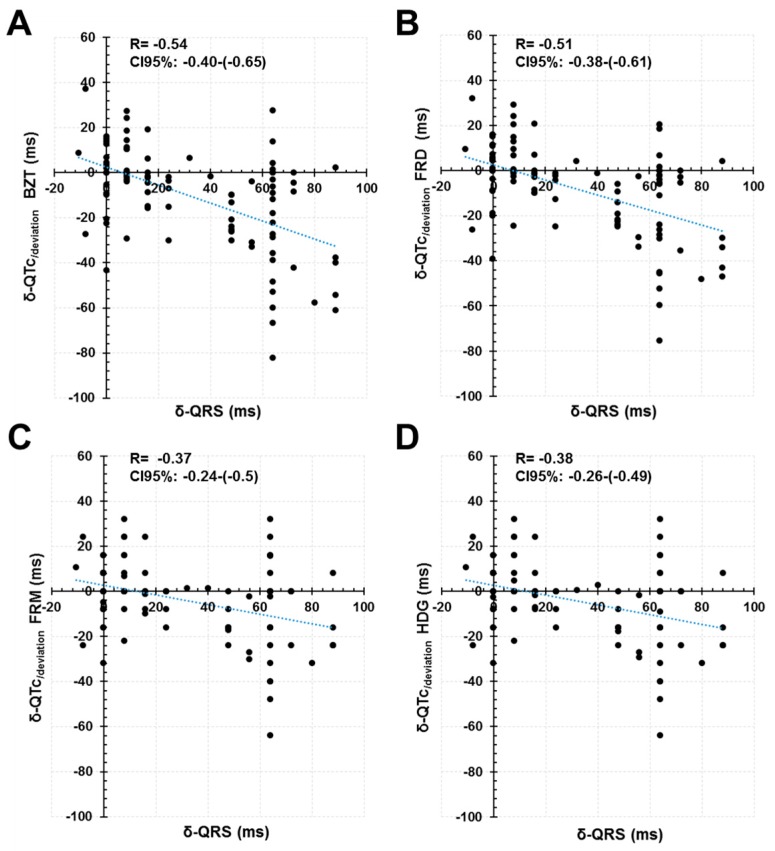
QRS dependence of the δ-QTc_/deviation_. See text for discussion. BZT: Bazett formula. FRD: Fridericia formula. FRM: Framinghan. HDG: Hodges formula.

**Figure 8:**
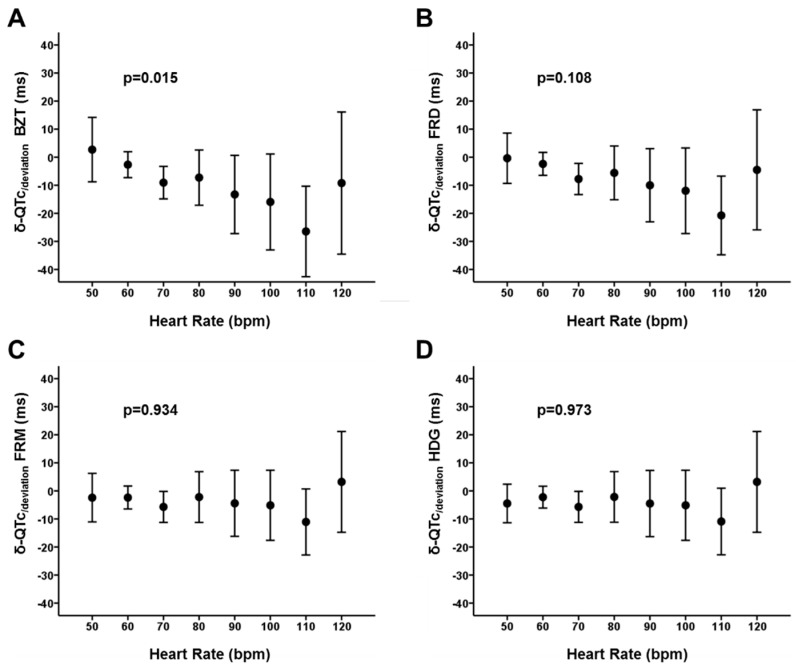
Heart rate dependence of the δ-QTc_/deviation_. See text for discussion. BZT: Bazett formula. FRD: Fridericia formula. FRM: Framinghan. HDG: Hodges formula.

Comparisons between phases (Figure 5, panel C) demonstrated that the magnitude of the δ-QTc_/deviation_ with the Bazett’s formula is higher than that obtained with the Fridericia, Framingham and Hodges formulas (*p* < 0.001). The correlation of the δ-QTc_/deviation_ with the δ-QRS seemed to be also higher with the Bazett’s formula (Pearson coefficient −0.54; *p* < 0.001) in comparison with the Fridericia (Pearson coefficient −0.51; *p* < 0.001), Framingham (Pearson coefficient −0.37; *p* < 0.001) and Hodges formulas (Pearson coefficient −0.38; *p* < 0.001) (Figure 7). Altogether, the data suggest that the Bazett’s formula displays significantly higher sensitivity than other formulas to prolongation of ECG intervals, decreasing the accuracy of measurements as the prolonged QRS (main contributor in our patients) also prolongs the length of the QT interval. There was also a significant effect of the heart rate in the δ-QTc_/deviation_ obtained with the Bazett’s formula (*p* = 0.015; Figure 8). However, the heart rate did not affect significantly the δ-QTc_/deviation_ with the Fridericia, Framingham and Hodges formulas (*p* = 0.108, 0.934 and 0.973 respectively; Figure 8)

## 4. Discussion

The main result of this study is that the δ-QRS explains most of the variation in the QT interval with prolonged depolarization of the ventricles. Other intervals like the time for repolarization and the extent of transmural dispersion of the repolarization contribute significantly with increased heart rate. We find that complex dynamic affects the accuracy of available formulas for frequency correction, leading the Framingham method the less sensitive to changes induced by the heart rate and the QRS widening.

### 4.1. Increased QRS Duration and Its Relationship with the QT Interval Dynamics

Cardiac repolarization is a complex, non-linear process, highly dependent on the coordination at multiple levels form the ionic domain in cardiac myocytes to the pattern and sequence of electrical propagation in the whole ventricle [16]. Prolonged intraventricular conduction alters the myocardial depolarization as well as the repolarization sequence. A recent consensus document pointed out that variations in the ST segment and T wave that occur as the direct result of changes in the sequence and/or duration of ventricular depolarization (QRS shape and/or duration), should be considered as secondary abnormalities in cardiac repolarization [2]. In other words, those changes in the ECG representation of cardiac repolarization are not the result of primary disorders affecting the physiology of the phase 2 and 3 of ventricular action potential in individual cells. But the changes in the sequence of depolarization alter the repolarization sequence, which theoretically may lead to two mayor phenomena explaining abnormal repolarization: first, the lack of simultaneous activation of the right and left ventricle (i.e., during bundle branch block); Secondly, the intensification of voltage gradients between myocardial layers and regions that are largely canceled during normal cardiac excitation. Those voltage gradients have been extensively studied in experimental conditions resembling different pathological conditions (i.e., Brugada syndrome and long QT syndrome) [17,18]. The electrotonic effect arising under electrical gradients may alter the physiology and shape of the action potential during its phase 2 and 3, explaining morphological changes in cardiac repolarization and arrhythmogenesis. Importantly, all those mechanisms are sensitive to the heart rate, which modulate the degree and functional repercussion of changes induced in cardiac repolarization [16]. Our study is the first experimental analysis of controlled heart rate comparing paired data from the same human with different QRS width. Changes in repolarization were controlled for biochemical, metabolic and hemodynamic conditions, which provide us with a unique opportunity to explore the role of variations in the QRS duration as a determinant of changes in cardiac repolarization and ECG intervals. Our data confirms that the QT interval linearly increase with prolongation of the QRS width and give credence to lack of simultaneous repolarization (as described above) as the main mechanisms explaining prolonged repolarization. But our data also suggest a direct effect on cardiac repolarization at the highest frequency range, which might be explained by the second mechanism proposed (intensification of voltage gradients). The latter might also explain the rate dependent variations observed within the ST segment and T wave (Figure 3).

Accordingly, some studies derived from population-based data suggested that the QT interval prolongs in ventricular conduction defects, and an adjustment for QRS duration becomes necessary [19,20,21]. Rautaharju PM et al. postulate that adjustment can be efficiently accomplished by incorporating QRS duration and RR interval as covariates into a QT-adjustment formula [20]. The same authors also observed that QT adjustment based on the simplify calculation of QT-QRS in patients with ventricular conduction defects retained a strong residual correlation with ventricular rate. They proposed that linear formulas with the analysis of the individual contribution of the QT and QRS intervals are desirable for making predictions as the method efficiently removed the rate dependence (QT_RR,QRS_ = QT − 155 × (60/heart rate-1) − 0.93 × (QRS-139) + *k*); with *k* = 22 ms for men and 34 ms for women) [20]. They also observed that if QTc-QRS is used in patients with ventricular conduction defects the correctly derived normal standards for some ECG intervals are not valid if the ventricular rate deviates from 60 bpm. Thus, the complex dynamic and interaction of factors is preventing direct conclusions by simply subtracting the QRS width from the length of the QT interval. Our data provide additional evidence for a rate dependence of changes in the repolarization that is adding complexity to the artifact introduced by the QRS widening.

### 4.2. Increased QRS Duration and Its Relationship with Frequency-Correction Formulas

From a clinical point of view, there is increasing interest in developing appropriate methods for computing QT intervals in patients with prolonged ventricle depolarization. In the last years, a variety of seminal works provide with different methods that allow for correction of QT intervals with regard to the QRS width [3,4,5]. However, after computing corresponding QT intervals, the frequency correction is still required. Here we pave the way for a comprehensive approach to dual correction (frequency plus QRS duration) and demonstrate that there is significant effect of QRS duration in the predictive capabilities of available formulas for frequency correction. All together we demonstrate that the Framingham formula is the least sensitive to the distortion introduced by prolonged depolarization, which will make it preferable in this particular scenario.

Consistent with previous studies, our data indicate that the diagnostic accuracy is different for the different correction formulas. The Bazett´s formula is the most dependent on the heart rate and tends to overestimate the QT interval when compared with others [15]. One of the reasons given for explaining these results is based on mathematical principles. The nonlinear formulas present exponential equations and therefore small variations in measurements increase the magnitude of the error provided. The extremes of the series (lower and higher frequency ranges) are the most affected values, which is consistent with our results. Simonson et al., [22] have already observed this behavior, which has been subsequently supported by numerous authors [23,24,25,26,27]. Chiladakis et al. [15] designed a model of controlled heart rate in patients with a dual chamber cardiac pacing device that preserved atrioventricular conduction and native QRS complexes with a duration of <120 ms. They compared the intrinsic conduction with the paced rhythms and evaluated the influence of heart rate on QTc through the use of various correction formulas. Their results confirmed that the Bazett’s formula is the most heart-rate dependent. We additionally demonstrate that the Bazett’s formula displays significantly higher sensitivity than other formulas to prolongation of ECG intervals, decreasing the accuracy of measurements as the prolonged QRS also prolongs the length of the QT interval. Accordingly, a recent work in patients with intraventricular conduction disorders propose the linear correction form Rautaharju et al. [20], which also provides with evidence for a comprehensive integration of the rate and QRS correction for the computing of QTc intervals [28]. We demonstrate that the dependence on the heart rate and QRS widening will not affect all the correction formulas uniformly; verifying that the loss of precision will be greater for the Bazett’s formula and that the linear formulas will be much less influenced (Framingham and Hodges). Overall, our results provide greater support for the use of linear formulas when attempting dual correction of the QT interval.

### 4.3. Limitations

The mechanism for QRS widening in our experimental protocol is related to intrahisian conduction abnormalities, which mostly promote intraventricular conduction defects resembling left bundle branch block [6,7]. We cannot ascertain that conclusions remain valid for other mechanisms prolonging ventricular depolarization (i.e., right bundle branch block, Purkinje’s fibers disease, depressed excitability/conduction, etc.). In addition, we studied a population with significant hypertrophy of the left ventricle, which has been associated with changes in the shape and duration of the ventricular action potential of isolated ventricular cells, particularly on the endocardial surface [29]. Although it is controversial to what extent such morphological changes in the repolarization sequence result in prolonged repolarization time, [19] we cannot rule out that hypertrophy influences results. The latter will limit the generalization of conclusions to patients with structurally normal hearts. In addition, the characteristics of cardiac depolarization (i.e., time course) within a frequency range may differ between spontaneous variations of the heart rate and our pacing model. Abrupt changes in the heart rate may lead to QRS widening. However, some authors demonstrated that the duration of the QRS complex is frequently shortened by spontaneous increases in heart rate [30,31] or burst pacing in the AAI mode [32]. The latest model is closer to our experimental protocol, but we fail to capture that dynamic behavior as the QRS width remained constant along our frequency range. However, the previous observation of QRS shortening with increased heart rate during atrial pacing [32] was mostly observed within a range of high frequencies above of the pacing limit stablished in our experimental setting. Thus, we were unable to explore that phenomena and how it affects results. As a consequence, validity of our results for the frequency range above 120 bxm should be taken with caution.

## 5. Conclusions

The prolonged depolarization of the ventricles introduces direct and linear prolongation in the QT interval, but also a non-linear distortion in cardiac repolarization that contributes for QT prolongation at the higher frequency range. That complex dynamic affects the accuracy of available formulas for frequency correction, leading the Framingham method the less sensitive to changes induced by the heart rate and the QRS widening.

## Figures and Tables

**Table 1:** Clinical characteristics of the patients included for analysis.

Characteristics			*N* = 23
Age (years)			84 (±4)
Weight (Kg)			70.29 (±12.98)
Height (cm)			158 (±8)
Body mass index			28.06 (±4, 9)
Male Gender (N; %)			11 (48%)
Cardiovascular Risk Factors			
	Hypertension (N; %)		20 (87%)
	Diabetes (N; %)		7 (30%)
	Dyslipidemia (N; %)		17 (74%)
	Smoking (N; %)		0 (0%)
		Former	5 (22%)
Coronary artery disease (N; %)			8 (35%)
Peripheral vascular disease (N; %)			6 (26%)
Chronic Kidney disease (N; %)			10 (44%)
	Dialysis treatment		0 (0%)
Echocardiographics findings			
	IVS (cm)		1.6 (±0.3)
	LVED Diameter (cm)		4,8 (±0.8)
	LV mass (g/m^2^)		167.76 (±55.18)
	RWT		0.59 (±0.19)
	LV ejection fraction (%)		56 (±14)
	Aortic mean gradient (mmHg)		50 (±14)
Antiarrhythmics			
	Class I (N; %)		0 (0%)
	Class II (N; %)		12 (52%)
	Class III (N; %)		0 (0%)
	Class IV (N; %)		1 (4%)
	Ivabradine (N; %)		1 (4%)

IVS: interventricular septum; LV: left ventricle; LVED: left ventricular end diastolic; RWT: relative wall thickness (posterior wall thickness^2^/LVED diameter).

**Table 2:** Comparative analysis of ECG morphology and measurements between phases.

		Baseline-Phase	Released-Phase	δ Released-Baseline (IC95%)	*p*-Value
QRS (ms)		98.50 (±21.78)	131.0 (±25.77)	32.5 (31.5–33.6)	*p* < 0.001
	Axis (degrees)	8.55° (±36.2)	3.18º (±43.64)		*p* = 0.376
	LBBB % (N)	0	39% (9)		*p* = 0.001
	RBBB % (N)	9% (2)	17% (4)		*p* = 0.162
	LAFH % (N)	13% (3)	13% (3)		
	LPFH % (N)	0	0		
QT (ms)		409.2 (±37.63)	444.6 (±45.41)	35.4 (33.4–37.4)	*p* < 0.001
50bpm		469.33 (±27.09)	520.0 (±17.53)		
60bpm		448.53 (±26.44)	477.16 (±37.80)		
70bpm		432.44 (±22.96)	468.0 (±31.79)		
80bpm		417.37 (±24.59)	454.48 (±32.06)		
90bpm		401.60 (±19.53)	435.60 (±34.58)		
100bpm		382.22 (±2.08)	417.33 (±33.97)		
110bpm		368.67 (±20.60)	404.67 (±30.60)		
120bpm		359.67 (±17.31)	388.0 (±31.42)		
QT_peak_ (ms)		322.2 (±32.12)	353.6 (±44.46)	31.3 (27.8–35.2)	*p* = 0.001
T_peak_-T_end_ (ms)		87.0 (±19.31)	91.07 (±28.35)	4.07 (−0.16–7.90)	*p* = 0.044
QRS_end_-T_peak_ (ms)		223.7 (±34.64)	222.5 (±43.92)	−1.18 (−4.84–2.61)	*p* = 0.563
QT_c_ Bazett’s (ms)		480.9 (±31.47)	522.5 (±4.21)	41.6 (38.6–44.5)	*p* < 0.001
QT_c_ Fridericia (ms)		454.5 (±23.92)	493.8 (±35.16)	39.3 (36.8–41.8)	*p* < 0.001
QT_c_ Framingham (ms)		449.2 (±22.35)	484.4 (±31.95)	35.3 (33.2–37.4)	*p* < 0.001
QT_c_ Hodges (ms)		453.6 (±21.65)	488.9 (±31.19)	35.4 (33.4–37.4)	*p* < 0.001

LBBB: left bundle branch block; RBBB: right bundle branch block; LAFH: left anterior fascicular hemiblock; LPFH: left posterior fascicular hemiblock.

**Table 3:** Univariate analysis of confounding factors.

	Baseline-Phase (SD)	Released-Phase (SD)	*p*-Value
Telesystolic LV presure (mmHg)	197.44 (±30.77)	159.61 (±33.65)	<0.001
Telediastolic LV presure mmHg	3.17 (±7.98)	17.06 (±17.85)	0.006
Temperature (°C)	36.77 (±0.4)	36.9 (±0.27)	0.047
Na^+^ (mmol/l)	141.61 (±3.09)	138.72 (±2.78)	0.053
K^+^ (mmol/l)	3.70 (±0.29)	3.70 (±0.37)	0.929
Ca^2+^ (mmol/l)	1.17 (±0.55)	1.12 (±0.52)	0.061
Cl^−^(mmol/l)	106.94 (±3.70)	106.17 (±3.94)	0.255
Glucose (mg/dl)	108.39 (±35.92)	107.56 (±34.23)	0.697
pH	7.37 (±0.33)	7.34 (±0.44)	0.049
pCO_2_ (mmHg)	46.83 (±6.03)	46.85 (±6.52)	0.981
HCO^3−^ (mmol/l)	27.32 (±3.25)	25.30 (±3.42)	0.051
Hemoglobin (g/dl)	11.76 (±1.60)	11.05 (±1.69)	0.069
Lactate (mmol/l)	0.55 (±0.22)	0.57 (±0.29)	0.959

**Table 4:** QTc_/deviation_ and the correlation coefficients with the QT60.

	QTc_/deviation_ * (ms; CI95%)		Correlation Coefficient with QT60 (CI95%)	
	Basaline-Phase	Released-Phase	Basaline-Phase	Released-Phase
Bazett	−31.1 (−35,0 – [−26.8])	−41.8 (−46.9 – [−36.4])	0.462 (0.344–0.574)	0.613 (0.505–0.706)
Fridericia	−6.46 (−8.61 – [−4.26])	−14.8 (−17.2 – [−12.4])	0.694 (0.628–0.755)	0.825 (0.783–0.862)
Framinghan	−1.14 (−3.38 – 1.19)	−5.42 (−7.51 – [−3.24])	0.698 (0.633–0.761)	0.843 (0.796–0.888)
Hodges	−5.05 (−6.78 – [−3.29])	−9.43 (−11.2 – [−7.70])	0.722 (0.651–0.789)	0.867 (0.836–0.897)

QTc: Corrected QT; * QTc_/deviation_ = QT measurement at 60 bpm − QTc.
